# Intravenous zoledronate for postmenopausal women with osteopenia and osteoporosis: a systematic review and metanalysis

**DOI:** 10.1590/1516-3180.2022.0480.R1.27032023

**Published:** 2023-05-26

**Authors:** Fernanda Martins Gazoni, Vinicius Tassoni Civile, Álvaro Nagib Atallah, Fânia Cristina Santos, Virginia Fernandes Moça Trevisani

**Affiliations:** I MD. Doctoral Student, Evidence-Based Health Program, Universidade Federal de São Paulo (UNIFESP), São Paulo (SP), Brazil; Geriatrician, Discipline of Geriatrics and Gerontology, Escola Paulista de Medicina (EPM), Universidade Federal de São Paulo (UNIFESP), São Paulo (SP), Brazil.; II MD, PhD. Physiotherapist, Evidence-Based Health Program, Universidade Federal de São Paulo (UNIFESP), São Paulo (SP), Brazil; Assistant Professor, Physiotherapy Course, Universidade Paulista (UNIP), São Paulo (SP), Brazil; Volunteer Researcher, Cochrane Brazil, São Paulo (SP), Brazil.; III MD, MSc, PhD. Nephrologist and Full Professor, Discipline of Emergency and Evidence-Based Medicine, Escola Paulista de Medicina (EPM), Universidade Federal de São Paulo (UNIFESP), São Paulo (SP), Brazil; Director, Cochrane Brazil, São Paulo (SP), Brazil.; IV MD, MSc, PhD. Geriatrician and Assistant Professor, Discipline of Geriatrics and Gerontology, Escola Paulista de Medicina (EPM), Universidade Federal de São Paulo (UNIFESP), São Paulo (SP), Brazil.; V MD, MSc, PhD. Rheumatologist and Assistant Professor, Discipline of Emergency and Evidence-Based Medicine, Escola Paulista de Medicina (EPM), Universidade Federal de São Paulo (UNIFESP), São Paulo (SP), Brazil; Rheumatologist and Full Professor, Discipline of Rheumatology, Universidade Santo Amaro (UNISA), São Paulo (SP), Brazil.

**Keywords:** Osteoporosis, Postmenopause, Fractures, bone, Zoledronic acid, Osteopenia, Systematic review, Metanalysis

## Abstract

**BACKGROUND:**

Osteoporosis compromises bone strength and increases the risk of fractures. Zoledronate prevents loss of bone mass and reduces the risk of fractures.

**OBJECTIVES:**

To determine the efficacy and safety of zoledronate in postmenopausal women with osteopenia and osteoporosis.

**DESIGN AND SETTINGS:**

A systematic review and meta-analysis was conducted within the evidence-based health program at the Universidade Federal de São Paulo.

**METHODS:**

An electronic search of the CENTRAL, MEDLINE, Embase, and LILACS databases was performed until February 2022. Randomized controlled trials comparing zoledronate with placebo or other bisphosphonates were included. Standard methodological procedures were performed according to the Cochrane Handbook and the certainty of evidence for the Grading of Recommendations Assessment, Development, and Evaluation Working Group. Two authors assessed the risk of bias and extracted data on fractures, adverse events, bone turnover markers (BTM), and bone mineral density (BMD).

**RESULTS:**

Twelve trials from 6,652 records were included: nine compared zoledronate with placebo, two trials compared zoledronate with alendronate, and one trial compared zoledronate with ibandronate. Zoledronate reduced the incidence of fractures in osteoporotic [three years: morphometric vertebral fractures (relative risk, RR = 0.30 (95% confidence interval, CI: 0.24–0.38))] and osteopenic women [six years: morphometric vertebral fractures (RR = 0.39 (95%CI: 0.25–0.61))], increased incidence of post-dose symptoms [RR = 2.56 (95%CI: 1.80–3.65)], but not serious adverse events [RR = 0.97 (95%CI: 0.91–1.04)]. Zoledronate reduced BTM and increased BMD in osteoporotic and osteopenic women.

**CONCLUSION:**

This review supports the efficacy and safety of zoledronate in postmenopausal women with osteopenia for six years and osteoporosis for three years.

**PROSPERO REGISTRATION NUMBER:**

CRD42022309708, https://www.crd.york.ac.uk/prospero/display_record.php?RecordID=309708.

## INTRODUCTION

Osteoporosis is a silent disease that compromises the density and quality of bones, increasing the risk of fractures. Advanced age and female sex are important risk factors for osteoporosis.^1^

Approximately 200 million women worldwide are observed to have osteoporosis,^2^ representing one-fifth of individuals over the age of 50 years.^3^ Although osteoporosis is responsible for a significant number of fractures, most fractures occur in individuals with osteopenia or with normal bone mineral density (BMD), which can be explained by the high number of people in this T-score range. Therefore, BMD results should be combined with other clinical risk factors for an accurate assessment of fracture risk and to guide treatment decisions.^4,5^ The most common sites where an osteoporotic fracture can occur are the vertebrae, hip, and distal forearm; however, the incidence of occurrence at other sites is also high.^6,7^

Drugs that increase bone mass do so by affecting bone metabolism. There are three categories: anti-catabolic (bisphosphonates, hormone therapy, selective estrogen-receptor modulators (raloxifene), and calcitonin), anabolic (teriparatide and abaloparatide), and both anabolic and anti-catabolic (romosozumab).^4^

Bisphosphonates are one of the first treatment choices for postmenopausal osteoporosis.^4,8^ They bind to hydroxyapatite in the bone mineral, inhibit the activity of osteoclasts, and prevent bone resorption.^9^

Zoledronic acid (or zoledronate) is an intravenous bisphosphonate that has a high affinity to the mineralized bone.^10^ It reduces the blood levels of bone turnover markers (BTM) (produced by osteoclasts) and increases bone mass (observed through densitometry).^10^ These findings are observed to correlate with a reduction in the number of new fractures.^10^

Prolonged use of bisphosphonates has been associated with complications, such as osteonecrosis of the jaw, excessive suppression of bone remodeling, atypical fractures of the femur, and atrial fibrillation.^11^

As the incidence of osteoporotic fractures continues to increase, global health demands therapies to reduce the risk of fractures. This systematic review helps to evaluate the evidence on the efficacy and safety of zoledronate in postmenopausal women, presenting an accessible updated synthesis to clinicians, researchers, health policy makers, and consumers, contributing to decision-making for preventing fractures.

## OBJECTIVE

To determine the efficacy and safety of zoledronate in postmenopausal women with osteopenia and osteoporosis.

## METHODS

The protocol was registered in PROSPERO (number CRD42022309708; https://www.crd.york.ac.uk/prospero/display_record.php?RecordID=309708).

### Study selection

All randomized controlled trials (RCTs) of a duration of at least one year comparing zoledronic acid (5 mg) to a placebo or other anti-catabolic agents in postmenopausal women were included. The inclusion criteria for RCTs for osteoporosis were: postmenopausal women with a previous fragility fracture and women with osteoporosis defined by densitometry (BMD T-score ≤ -2.5 standard deviation [SD]), with or without previous fragility fractures; and for osteopenia were: postmenopausal women without fragility fractures and with a T-score < -1 SD and > -2.5 SD. Trials that investigated women with secondary osteoporosis (bone loss caused by specific diseases, including malignancy, or medications) were excluded.

### Search methods for the identification of studies

On May 13, 2021, and February 15, 2022, electronic databases, such as the Cochrane Central Register of Controlled Trials (CENTRAL), MEDLINE (via PubMed); EMBASE (via Ovid), and LILACS (Latin American and Caribbean Health Science Information database) were searched for all relevant RCTs, regardless of language or publication status. In addition, trial registers for study protocols, ongoing trials, and conference abstracts were searched.

### Study outcomes

The primary outcomes were fractures and adverse reactions each year. The fractures were classified as follows: incidence of clinical and morphometric vertebral fractures, non-vertebral fractures, hip fractures, and all fractures. For adverse reactions, the following were considered: non-serious and serious adverse events (SAE), total mortality, atrial fibrillation, post-dose symptoms or influenza-like symptoms, increase in serum creatinine (a rise of more than 0.5 mg per deciliter (or 44 μmol/L) compared with the baseline level), osteonecrosis of the jaw, atypical femoral fractures, and eye disorders (uveitis, iritis, episcleritis).

The secondary outcomes were percent change in BTM, such as CTX (C-terminal telopeptide of type 1 collagen) and P1NP (Procollagen type 1 N propeptide) after 6 months and after each year, and percent change in BMD of the lumbar spine, femoral neck, and total hip after each year.

### Data collection

Data were extracted systematically in a predefined and standardized manner according to the instructions given in the Cochrane Handbook for Systematic Reviews of Interventions.^12^ Two review authors independently selected the studies that matched the inclusion criteria, screened titles and abstracts, selected reports to read in full text, and independently extracted all data from the studies. In addition, the risk of bias was assessed using domain-based evaluation criteria and was judged as low, with some concerns, or a high risk of bias. When necessary, a third reviewer was consulted to settle any disagreements.^12^

### Data analysis

Risk ratios (RR) were calculated for dichotomous variables with 95% confidence intervals (CI), and the relative percent change was calculated and expressed as a percentage. The number needed to treat for a benefit or the number needed to cause harm was calculated for significant outcomes.^12,13^ The mean difference (MD) in the percent change from baseline with 95% CI was calculated for continuous data.^12,13^ The WebPlotDigitizer program (https://github.com/ankitrohatgi/WebPlotDigitizer, version 3, Pacifica, California, United States) was used to extract values from graphics when the data were not available in the text.^14^

Meta-analyses were performed in a random-effects model to avoid ’between-study’ variations when the data were clinically and statistically homogeneous, as recommended in the Cochrane Handbook for Systematic Reviews of Interventions.^13^ Heterogeneity was assessed using the chi-square test (with the significance set at a P value of 0.05) and measured through I^2^ (I^2^> 50% was considered to signify substantial heterogeneity).^13^

The overall certainty of the evidence was independently assessed by two authors using the specific evidence grading system developed by the Grading of Recommendations Assessment, Development, and Evaluation (GRADE) Working.^15^ The GRADE approach specifies four levels of certainty of evidence (high, moderate, low, or very low).

A minimum significant reduction value for fractures was established in this review to consider zoledronate effective. The values varied according to the fracture type: 30% for vertebral and hip fractures, 15% for non-vertebral fractures and all clinical fractures.^16^ Any increase in serious adverse events or a 10% increase in non-serious adverse events was considered significant.^16^

A minimal significant reduction in the BTM levels (CTX and P1NP) of 30%, a minimal significant increase in the BMD values measured by dual-energy X-ray absorptiometry of 5% at the lumbar spine, and a 4% increase at the femoral neck and total hip were considered.^16^

## RESULTS

### Results of the search

**Figure 1.**
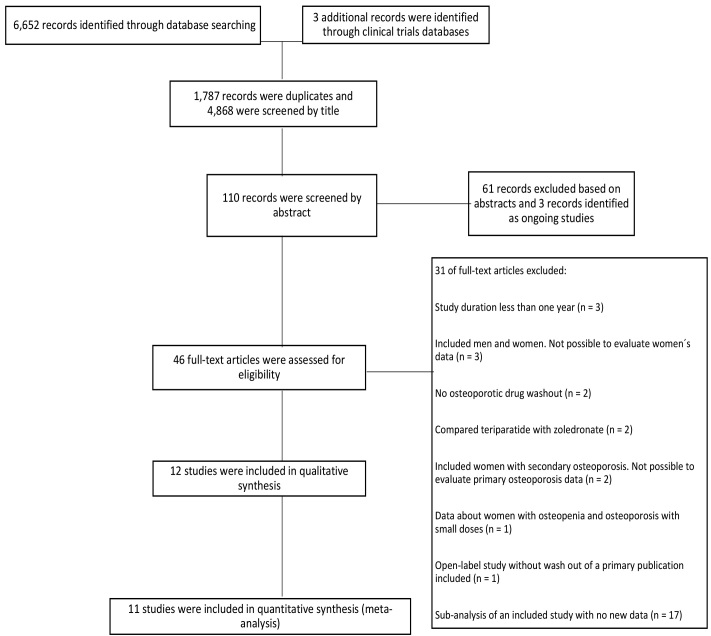
Preferred Reporting Items for Systematic Reviews and Meta-Analyses (PRISMA) flow diagram.

A total of 6,652 records were identified. After removing duplicates (1,787 records), 12 RCTs met the eligibility criteria,^17–29^ and 11 RCTs were included in the meta-analysis (Figure 1). One RCT published data in more than one article.^17,18^ Six RCTs compared zoledronate yearly with placebo,^17–23^ two RCTs compared zoledronate with alendronate,^27,28^ one RCT compared zoledronate with ibandronate,^29^ two RCTs studied a single dose,^24,25^ and one RCT investigated each 18-month period over six years.^26^ The characteristics of the included studies are summarized in Table 1 ^17,19–22,27–29^ for osteoporosis and Table 2 ^23–26^ for osteopenia.

**Table 1.** Summary of characteristics of included studies with osteoporotic women

Study ID	Study duration (years)	Comparator	Number of participants	Ethnicity	Inclusion criteria	Age (years)	Outcomes	Industry funding
Bai et al.,^20^ 2013	2	0.25 mg activated vitamin D3	242 Zol X 241 Plac	Chinese	low bone mass + fracture or osteoporosis	Zol: 56.5 ± 6.83 Plac: 57.15 ± 6.3	Fractures AEs BMD	No
Black et al.,^17^ 2007	3	Placebo	3,875 Zol X 3,861 Plac	More than 15 countries	low bone mass + fracture or osteoporosis	Zol: 73.0 ± 5.2 Plac: 73.1 ± 5.4	Fractures AEs BTM BMD	Yes
Chao et al.,^19^ 2013	1	0.25 mg activated vitamin D3	327 Zol X 333 Plac	Chinese	low bone mass + fracture or osteoporosis	Zol: 54.6 ± 7.3 Plac: 55.3 ± 7.5	Fractures AEs	Yes
Liang et al.,^21^ 2017	2	Placebo	175 Zol X 110 Plac	Chinese	only osteoporosis by DXA	Zol: 57.22 ± 2.8 Plac: 57.48 ± 3.2	Fractures BTM BMD	Yes
Yang et al.,^22^ 2015	1	Placebo	50 Zol X 50 Plac	Chinese	only osteoporosis by DXA	Zol:61.4 ± 9.5 Plac: 59.7 ± 8	BTM BMD	No
Hadji et al.,^28^ 2012	1	Alendronate 70 mg/week	408 Zol X 191 Aln	Germany	only osteoporosis by DXA	Zol: 67.6 ± 8.0 Aln: 68.1 ± 7.9	Fractures (AE) AEs BTM	Yes
Tan et al.,^27^ 2016	3	Alendronate 70 mg/week	52 Zol X 53 Aln	Chinese	only osteoporosis by DXA	Zol: 68.1 ± 9.02 Aln: 68.0 ± 8.55	Fractures (AE) BTM BMD	No
Gonnelli et al.,^29^ 2014	1	Ibandronate 3 mg/3 months	30 Zol X 30 Ibn	Italian	low bone mass + fracture or osteoporosis	Zol: 64 ± 6 Ibn: 67.0 ± 8.1	BTM BMD	No

Zol = Zoledronate; Plac = Placebo; Aln = Alendronate; Ibn = Ibandronate; DXA = dual-energy x-ray absorptiometry (DXA) or bone densitometry; AEs = adverse events; BTM = bone turnover marker; BMD = bone mineral density.

**Table 2.** Summary of the characteristics of included studies with osteopenic women

Study ID	Study duration (years)	Comparator	Number of participants	Ethnicity	Inclusion criteria	Age (years)	Outcomes	Industry funding
Grey et al.,^24^ 2009	2	Placebo	25 Zol X 25 Plac	New Zealand	low bone mass + no fractures	Zol: 62 ± 8 Plac: 65 ± 8	Fractures (AE) AEs BTM BMD	No
Grey et al.,^25^ 2012	1	Placebo	43 Zol X 43 Plac	New Zealand	low bone mass + no fractures	ZOL: 66 ± 8 Plac: 65 ± 9	Fractures (AE) AEs BTM BMD	No
McClung et al.,^23^ 2009	2	Placebo	379 Zol X 202 Plac	25 centers	low bone mass + no fractures	Zol: 59 ± 8 Plac: 60 ± 8	Fractures (AE) AEs BTM BMD	Yes
Reid et al.,^26^ 2018	6	Placebo	1,000 Zol X 1,000 Plac	New Zealand	low bone mass + no fractures	Zol: 71 ± 5 Plac: 71 ± 5	Fractures AEs BTM BMD	No

Zol = Zoledronate; Plac = Placebo; DXA = dual-energy x-ray absorptiometry (DXA) or bone densitometry; AEs = adverse events; BTM = bone turnover marker; BMD = bone mineral density.

### Risk of bias in included studies

**Figure 2.**
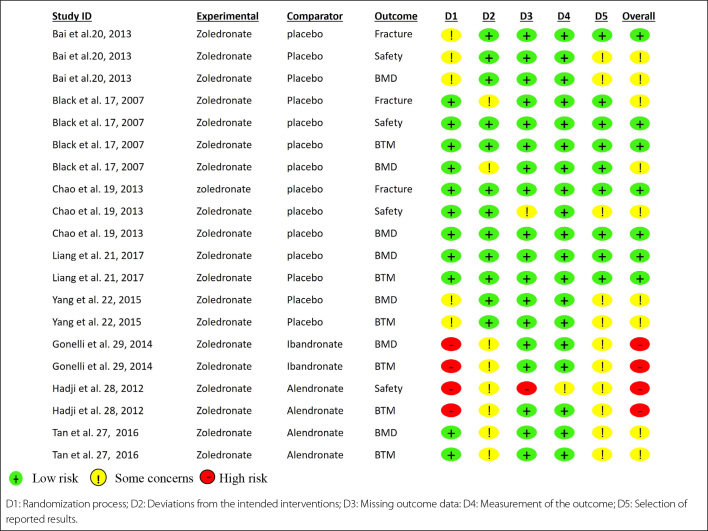
Risk of bias 2: Risk of bias for each outcome in all randomized controlled trials with osteoporotic women. D1: Randomization process; D2: Deviations from the intended interventions; D3: Missing outcome data: D4: Measurement of the outcome; D5: Selection of reported results.

**Figure 3.**
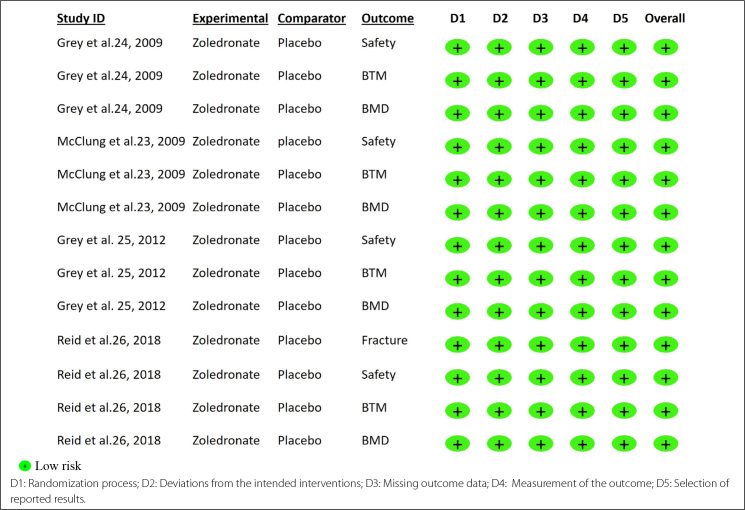
Risk of bias 2: risk of bias for each outcome in all randomized controlled trials with osteopenic women. D1: Randomization process; D2: Deviations from the intended interventions; D3: Missing outcome data; D4: Measurement of the outcome; D5: Selection of reported results. Low risk

The risk of bias for each study and outcome is shown in Figure 2 for osteoporosis and Figure 3 for osteopenia, respectively.

### Primary outcomes

#### Fractures

The incidence of fracture data was obtained from four RCTs comparing zoledronate with a placebo in women with osteoporosis and one RCT conducted on women with osteopenia. The RCTs comparing zoledronate with alendronate reported fractures as adverse events, whereas the RCT comparing ibandronate did not evaluate fractures.

#### Postmenopausal osteoporotic women

**Figure 4.**
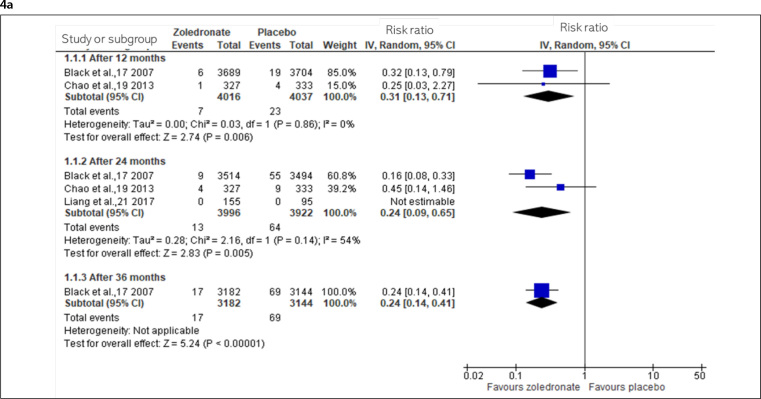

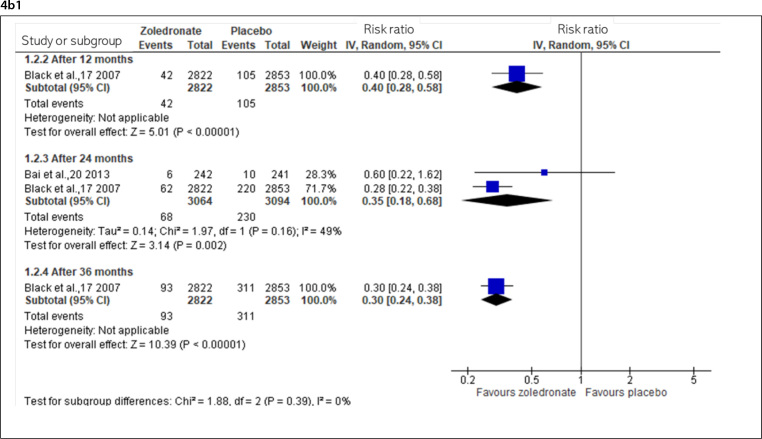

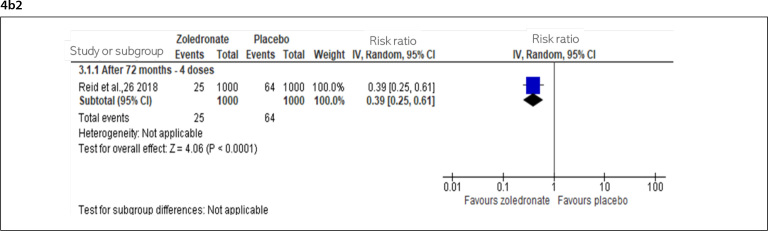

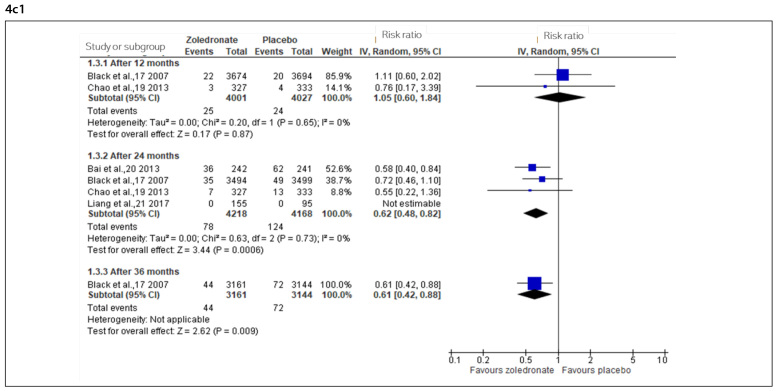

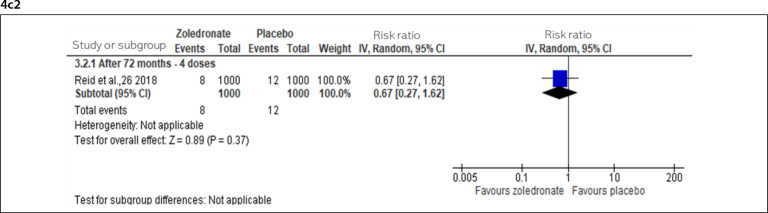

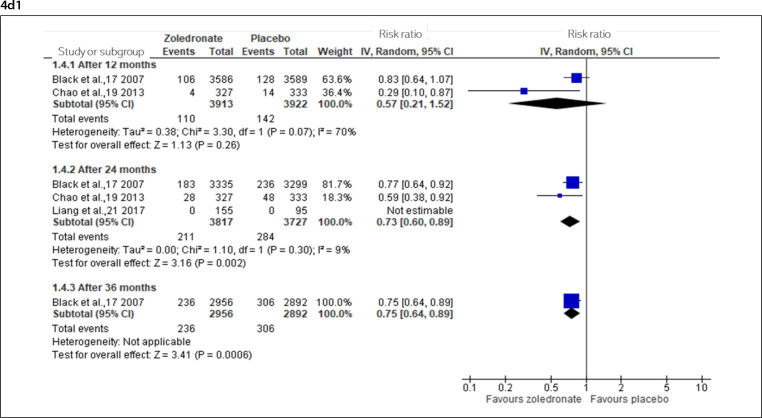

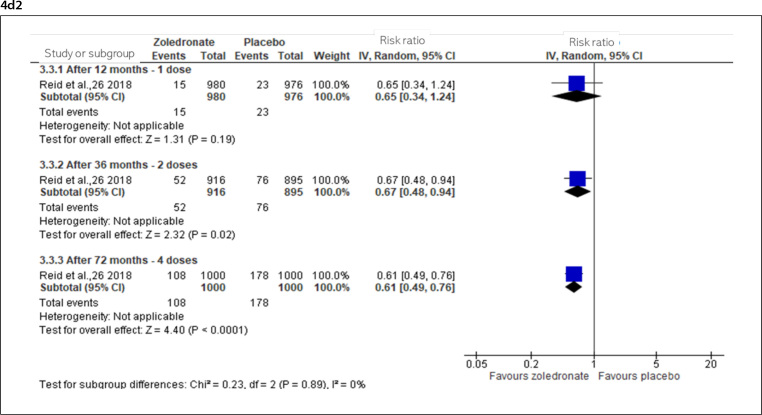

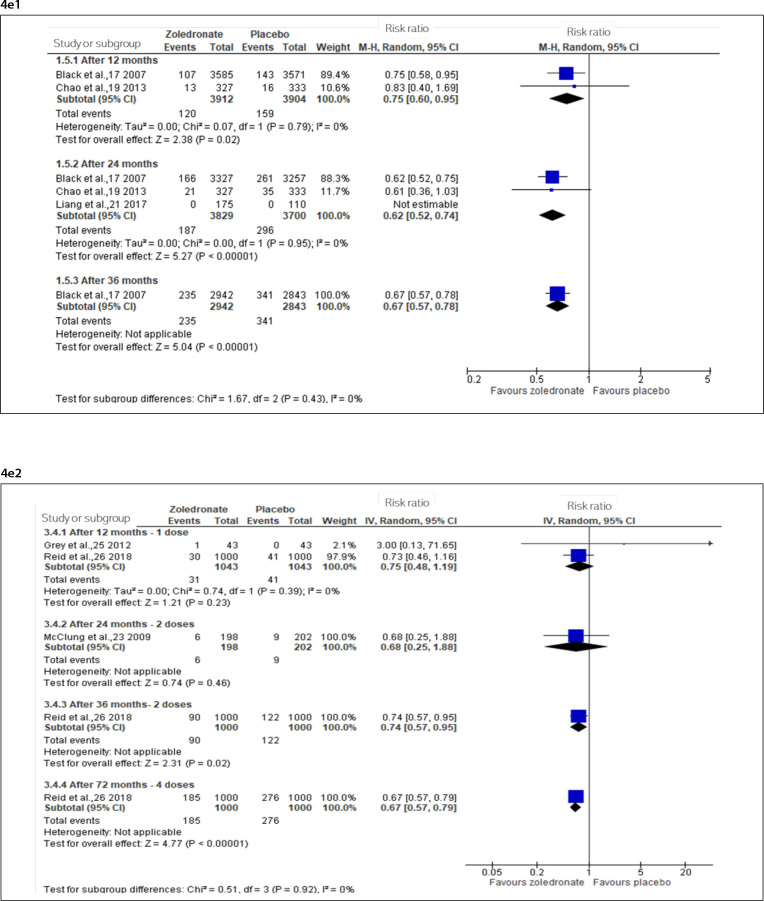
Incidence of fractures from 12 to 72 months in osteoporotic and osteopenic women [4a: Vertebral fractures (osteoporotic); 4b: Morphometric vertebral fractures (4b1: osteoporotic; 4b2: osteopenic), 4c: Hip fractures (4c1: osteoporotic; 4c2: osteopenic), 4d: Non-vertebral fractures (4d1: osteoporotic; 4d2: osteopenic), and 4e: All clinical fractures (4e1: osteoporotic; 4e2: osteopenic)].

Upon comparing zoledronate with placebo, high-certainty evidence demonstrating that zoledronate reduces clinical and morphometric vertebral fractures since the first year was obtained (Figure 4a and Figure 4b1).

For hip fractures (Figure 4c1), zoledronate had no effect on reducing or increasing hip fractures after one year; however, moderate-certainty evidence (downgraded for imprecision) indicated that zoledronate probably reduces hip fractures after two years.

There was also moderate-certainty evidence (downgraded for imprecision) that zoledronate probably reduces non-vertebral fractures after two and three years (Figure 4d1) and high-certainty evidence that zoledronate reduces all clinical fractures after two and three years (Figure 4e1).

Upon comparing zoledronate with alendronate after one year, there was very low-certainty (downgraded by two points for risk of bias and one for imprecision) about the effect of zoledronate on hip fractures and clinical fractures.

#### Postmenopausal osteopenic women

High-certainty evidence indicated that 5 mg of zoledronate every 18 months reduces morphometric vertebral fractures after six years (four doses) (Figure 4b2).

For hip fractures (Figure 4c2), moderate-certainty evidence (downgraded for imprecision) indicated that zoledronate probably results in little to no difference in the reduction of hip fractures after six years (four doses).

For non-vertebral fractures (Figure 4d2), moderate-certainty evidence (downgraded for imprecision) indicated that zoledronate likely results in little to no difference in preventing non-vertebral fractures after one year; however, after three years (2 doses), zoledronate probably reduces and after six years (4 doses), there is a high certainty that it reduces non-vertebral fractures.

For all clinical fractures, moderate-certainty evidence (downgraded for imprecision) indicated that zoledronate probably results in little to no difference in preventing clinical fractures in the first two years (Figure 4e2); however, after three years (2 doses), moderate-certainty evidence (downgraded for imprecision) indicated that zoledronate likely reduces clinical fractures. Additionally, after six years (4 doses), high-certainty evidence indicated that it reduces clinical fractures.

#### Adverse reactions

The incidence of adverse events was obtained from seven RCTs comparing zoledronate with placebo and one RCT comparing zoledronate with alendronate. An RCT of ibandronate did not evaluate any adverse events.

**Figure 5:**
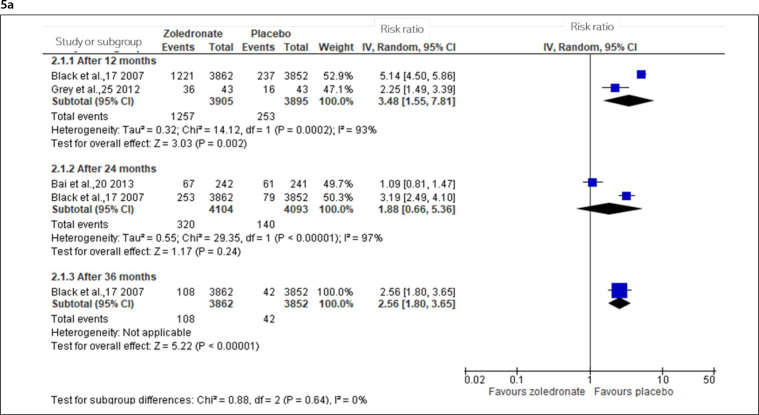

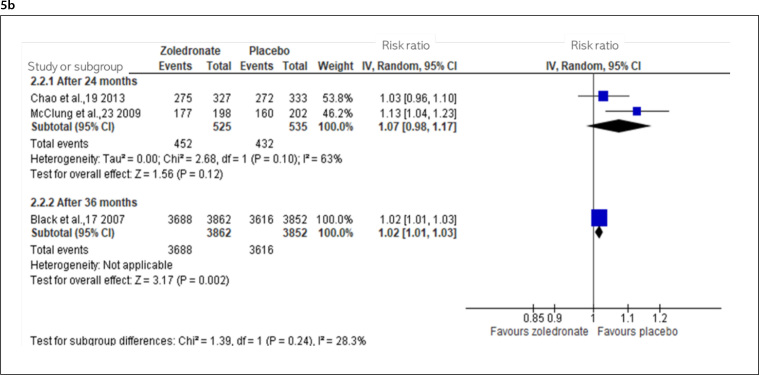

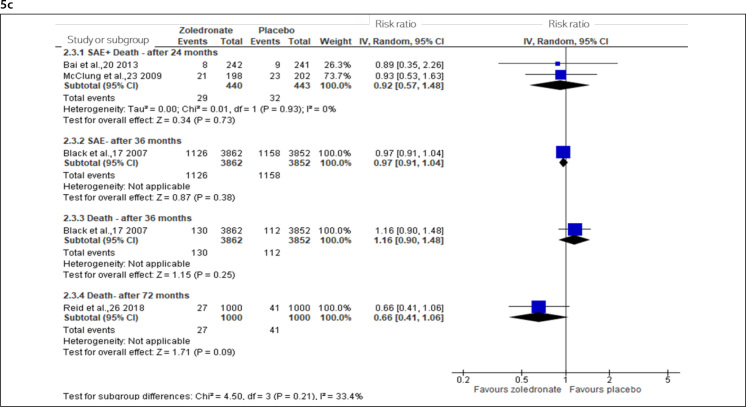

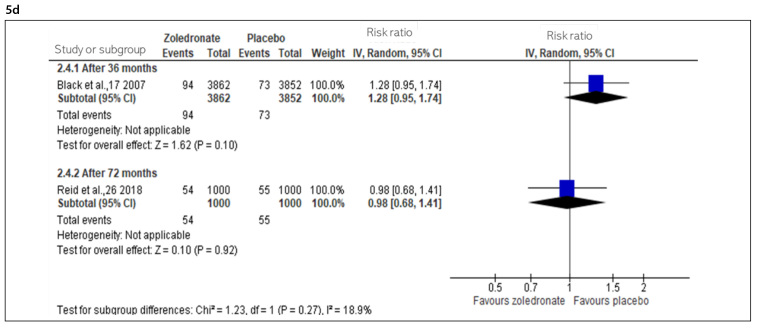
Incidence of adverse events from 12 to 72 months in osteoporotic and osteopenic women (5a: Any symptom of post-dose acute-phase reactions, 5b: Non-serious adverse events, 5c: Serious adverse event or death, and 5d: Atrial fibrillation).

A comparison of zoledronate with placebo showed that moderate- to high-certainty evidence indicated that zoledronate increases the post-dose symptoms after one year (Figure 5a). Low-certainty evidence (downgraded for risk of bias and inconsistency) indicated that zoledronate may increase the post-dose symptoms after two years, and high-certainty evidence indicated that zoledronate may increase the post-dose symptoms after three years.

After two years, moderate-certainty evidence indicated that zoledronate may slightly increase non-serious adverse events (Figure 5b), and after three years, high-certainty evidence indicated that zoledronate did not increase non-serious adverse events.

Moderate-certainty evidence (downgraded for imprecision) indicated that zoledronate probably results in no difference in the SAE or death after two years (Figure 5c). After three years, moderate-certainty (downgraded for imprecision) indicated that it probably does not reduce or increase the SAE or death, and after six years (four doses), moderate-certainty evidence (downgraded for imprecision) indicated that zoledronate probably results in no difference in death.

After three years, moderate-certainty evidence (downgraded for imprecision) indicated that zoledronate may slightly increase the atrial fibrillation; but after six years (four doses), zoledronate probably does not increase atrial fibrillation (Figure 5d).

Moderate-certainty evidence (downgraded for imprecision) indicated that zoledronate probably results in little to no difference in eye disorders after one year, and after three years, it probably does not increase jaw osteonecrosis. After three years, high-certainty evidence indicated that the serum creatinine levels has increased.

In a study comparing zoledronate with alendronate, low-certainty evidence (downgraded for risk of bias and imprecision) indicated that zoledronate increases adverse events and influenza-like symptoms, results in little to no difference in serious adverse events or death, and does not increase or reduce atrial fibrillation or eye disorders after one year.

### Secondary outcomes

#### Percent change in bone turnover markers (BTM)

The percent change in BTM was obtained from six RCTs that compared zoledronate with a placebo. Yang et al. also analyzed the BTM; however, the data from this RCT were not used in the review because the baseline values were different when compared to others.^22^

#### Postmenopausal osteoporotic women

**Figure 6:**
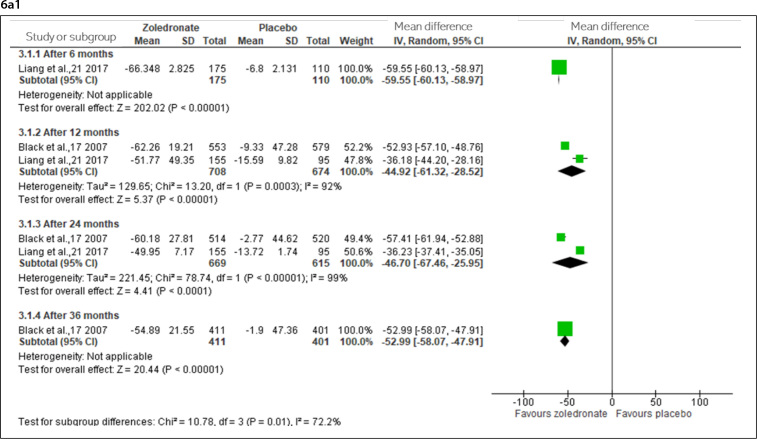

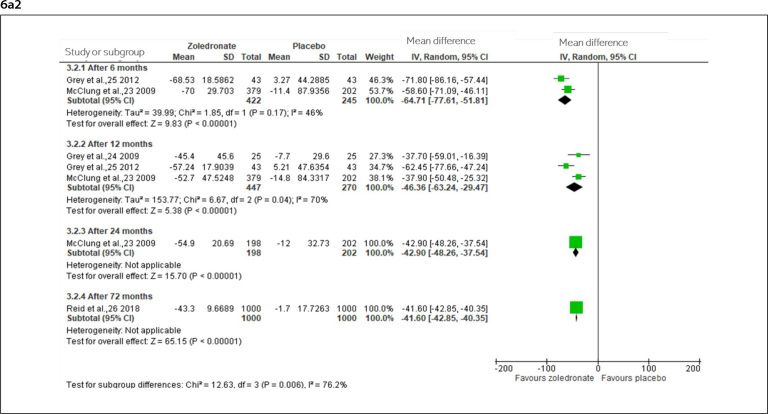

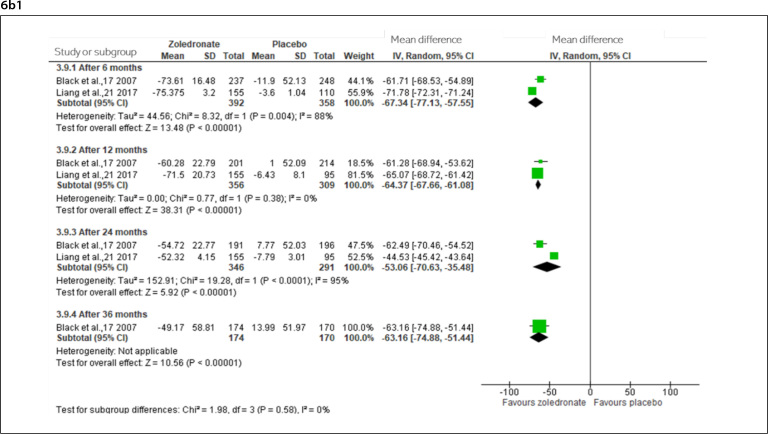

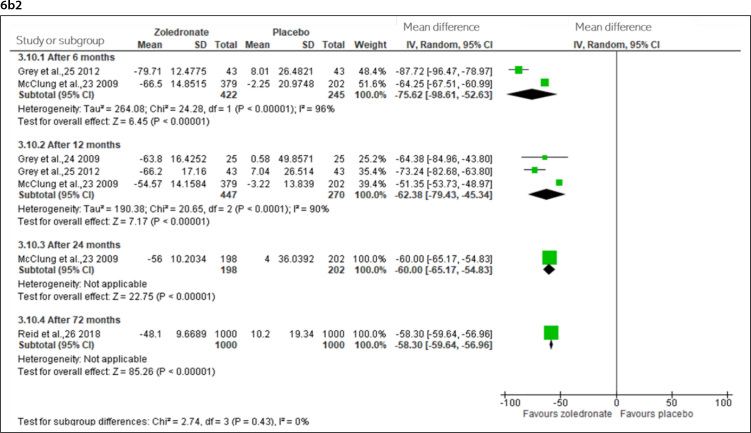
Percent change in bone turnover markers from 6 to 72 months in osteoporotic and osteopenic women [6a- Procollagen type 1 N propeptide (6a1- osteoporotic; 6a2- osteopenic), and 6b- C-terminal telopeptide of type 1 collagen (6b1- osteoporotic; 6b2- osteopenic)].

After six months, moderate-certainty evidence (downgraded for imprecision) indicated that zoledronate probably reduces P1NP. After one and two years, the certainty was low (downgraded for inconsistency and imprecision), and after three years, high-certainty evidence indicated that zoledronate reduces P1NP (Figure 6a1).

After six months and one year, high-certainty evidence indicated that zoledronate reduces the CTX levels. After two and three years, the evidence was moderate (downgraded for imprecision) (Figure 6b1).

Low-certainty evidence (downgraded for the risk of bias and imprecision) indicated that zoledronate results in little to no difference in P1NP and that it probably reduces the CTX compared to alendronate. For zoledronate versus ibandronate, very low-certainty evidence (downgraded by one point for risk of bias and two points for imprecision) indicated that zoledronate has no effect on CTX.

#### Postmenopausal osteopenic women

After six months, high-certainty evidence indicated zoledronate reduces P1NP, and after one year, low-certainty evidence (downgraded for inconsistency and imprecision). After two years (two doses) and six years (four doses), high-certainty evidence indicated that zoledronate reduces P1NP (Figure 6a2).

After six months and one year, moderate-certainty evidence (downgraded for inconsistency) indicated that zoledronate reduces the CTX. High-certainty evidence was observed after two years (two doses) and six years (four doses) (Figure 6b2).

#### Percent change in BMD

The MD in BMD was obtained from eight RCTs that compared zoledronate with placebo, one with alendronate, and one with ibandronate.

#### Postmenopausal osteoporotic women

**Figure 7.**
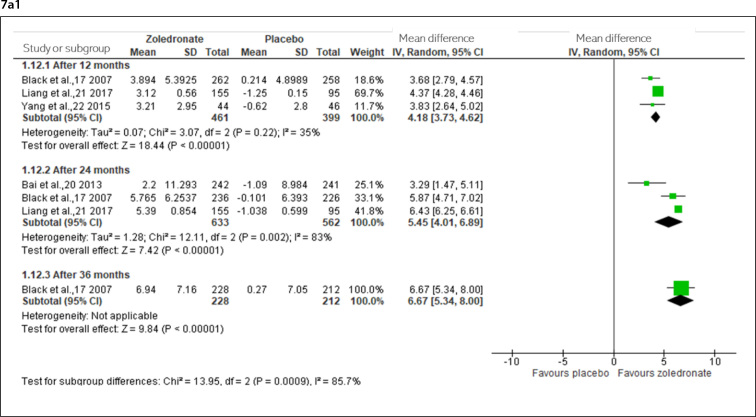

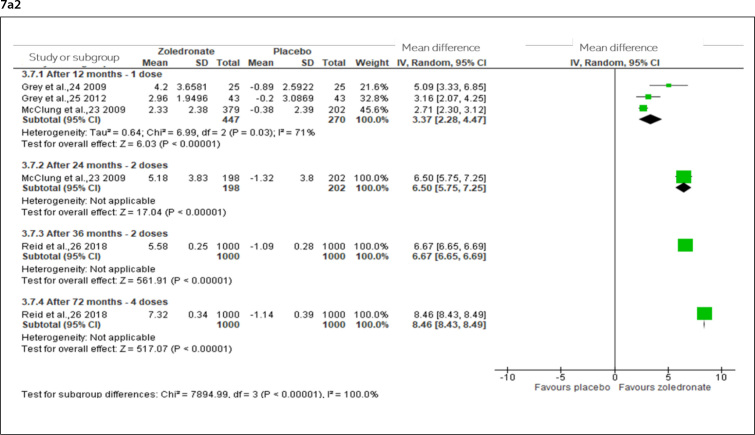

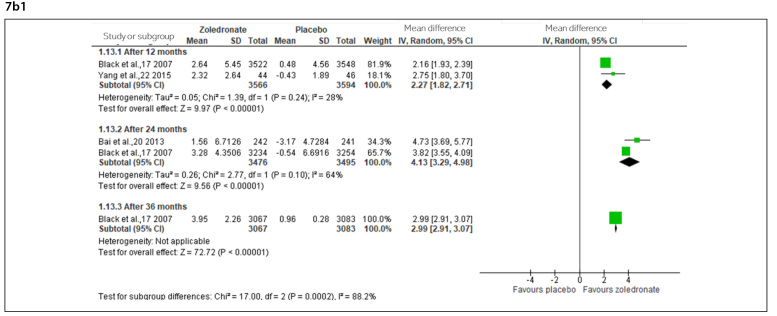

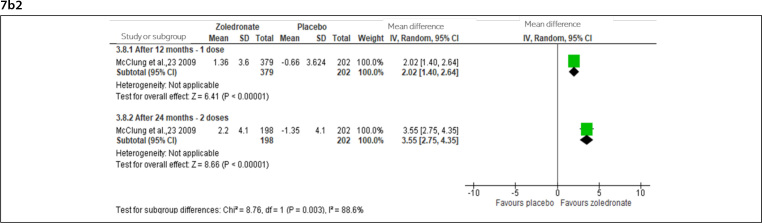

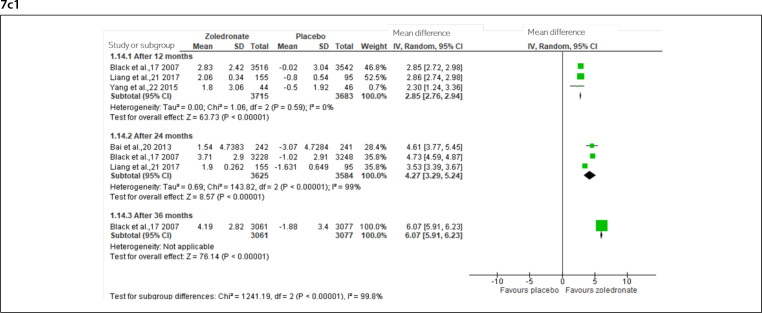

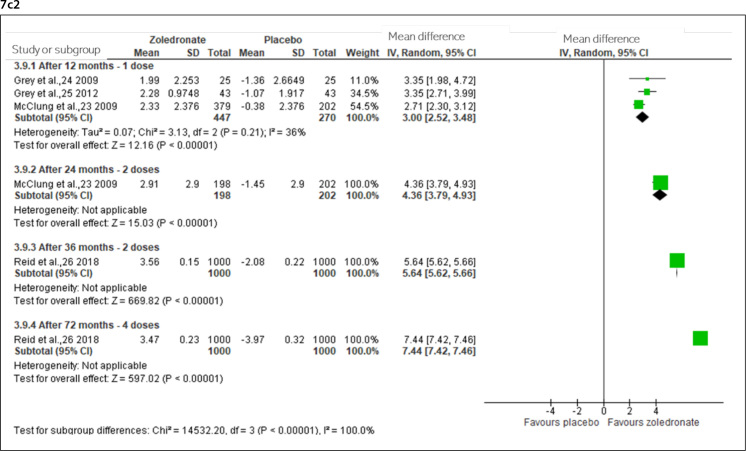
Percent change in bone mineral density from 12 to 72 months in osteoporotic and osteopenic women [7a- lumbar spine (7a1- osteoporotic; 7a2- osteopenic), 7b- femoral neck (7b1- osteoporotic; 7b2- osteopenic), and 7c- total hip (7c1- osteoporotic; 7c2- osteopenic)].

Moderate-certainty evidence (downgraded for risk of bias) indicated that zoledronate probably does not increase the lumbar spine BMD after one year; however, it was observed to probably increase after two years and increase after three years (Figure 7a1).

Moderate-certainty evidence (downgraded for risk of bias) indicated that zoledronate probably does not increase the femoral neck BMD after one year and three years, and low-certainty evidence (downgraded for inconsistency and imprecision) probably results in little increase after two years (Figure 7b1).

Moderate-certainty evidence (downgraded for risk of bias) indicated that zoledronate probably does not increase the total hip BMD after one year, may increase after two years, and that it increases after three years (Figure 7c1).

For zoledronate versus alendronate, low-certainty evidence (downgraded for risk of bias and imprecision) indicated that zoledronate increases lumbar spine, femoral neck, and total hip BMD. For zoledronate versus ibandronate, very low-certainty evidence (downgraded by one point for risk of bias and two points for imprecision) indicated uncertainty about the presence of an effect on the lumbar spine and total hip BMD.

#### Postmenopausal osteopenic women

Moderate-certainty evidence (downgraded for inconsistency) indicated that zoledronate probably does not increase the lumbar spine BMD after one year. After two years (two doses), three years (two doses), and six years (four doses), there was high-certainty evidence that zoledronate increases the lumbar spine BMD (Figure 7a2).

High-certainty evidence indicated that zoledronate does not increase the femoral neck BMD after one year, and moderate-certainty evidence (downgraded for imprecision) indicated that it results in little to no difference in increasing the femoral neck BMD after two years (Figure 7b2).

High-certainty evidence indicated that zoledronate does not increase the total hip BMD after one year, and moderate-certainty (downgraded for imprecision) indicated that it may increase the total hip BMD after two years; however, after three years (two doses) and six years (four doses), high-certainty evidence indicated that it increases total hip BMD (Figure 7c2).

## DISCUSSION

In this systematic review, 12 RCTs on the use of zoledronate in postmenopausal women were included: eight RCTs for osteoporosis and four RCTs for osteopenia. To assess whether there is an effective and safe response to zoledronate, thresholds of statistical significance were established according to published data found in the scientific literature.

The main objective for preventing and treating osteoporosis is to reduce the incidence of fractures, although it does not eliminate them. In this review, a minimal significant reduction of 30% was established for vertebral and hip fractures, and a 15% reduction was established for other fractures (non-vertebral and clinical). This decision was based on the thresholds for therapeutic failure published by Diez-Perez et al.^16^ They considered a reduced risk of fractures ranging from 30% to 70% for vertebral fractures, 40% to 50% for hip fractures, and 15% to 20% for non-vertebral fractures.^16^

The occurrence of fractures in the RCTs was evaluated as both an outcome and an adverse event, which could have influenced the results of the analyses. The evidence for fractures was moderate to high, indicating that zoledronate reduces clinical and morphometric vertebral fractures since the first year of use, increasing its benefits each year during 3 years of treatment for osteoporotic women and for six years (5 mg every 18 months) for osteopenic women. In addition, zoledronate probably reduces the number of hip fractures after two years in osteoporotic women and probably results in little difference after six years (5 mg every 18 months) in osteopenic women. Zoledronate probably reduces non-vertebral fractures after two doses in women with osteoporosis (5 mg each year) and after two doses (5 mg every 18 months) and four doses (six years) in women with osteopenia. It reduces the number of all clinical fractures after two doses in both osteoporotic (after two years) and osteopenic (after three years) women and after six years (four doses) for osteopenic women.

No data were available regarding ibandronate-related fractures. Compared to alendronate, the results were based on fractures reported as adverse events, and the evidence was of very low certainty.

Post-dose symptoms were reported mainly after the first infusion but also after the third dose. This was expected because, according to a literature review, acute-phase reactions can occur in up to 30% of patients.^30^

There were no statistically significant differences with respect to serious adverse events, death, atrial fibrillation, osteonecrosis of the jaw, or eye disorders between the zoledronate and placebo groups. Osteonecrosis of the jaw has been described in cancer patients receiving high doses of intravenous zoledronate; however, its incidence in osteoporotic patients treated with zoledronate is considered very low.^31^ Additionally, concerns have been raised regarding the possible association between bisphosphonate therapy and atrial fibrillation. A meta-analysis of RCTs and observational studies with women and men treated with bisphosphonates for any indication demonstrated an increased risk of atrial fibrillation with bisphosphonates (slightly higher with intravenous bisphosphonates).^32^ Eye disorders, although rare, were associated with all bisphosphonate treatments.^33^ Patel et al. reported an incidence of uveitis and episcleritis of 1.1% (95% CI 0.5–2.1).^34^

None of the RCTs included in this review reported atypical femoral fractures. Atypical femoral fractures of the subtrochanteric region are considered rare events; however, bisphosphonate treatment for more than five years increases the risk of such fractures.^35^

One RCT found a risk of increasing serum creatinine after zoledronate infusion.^17^ This effect was noted by the U.S. Food and Drug Administration in 2011, advising no use in patients with creatinine clearance less than 35 mL/min or with acute renal impairment and monitoring of renal function in patients receiving zoledronic acid.^36^

A position paper endorsed by the International Osteoporosis Foundation published that a significant response to antiresorptive treatments occurs when there is a decline from baseline of at least 25% for CTX and P1NP.^16^ Delmas et al. reported that a decrease in the BTM could range from 30 to 50% after starting treatment with bisphosphonates.^37^ A reduction of at least 30% in BTM was also found in the data presented in this review.

The same study reported that the least significant change in BMD should be approximately 5% in the lumbar spine and 4% in the femoral neck.^16^ A meta-regression analysis reported that a 4% increase in BMD of the femoral neck and total hip reduced vertebral fractures by 50% and hip fractures by 30%, and an increase in the lumbar spine BMD of 2% and 8% reduced vertebral fractures by 30% and 60%, and hip fractures by 20% and 40%, respectively.^38^ Therefore, the present review considered a least significant change of a 5% increase in the lumbar spine and 4% in the femoral neck and total hip. Based on these thresholds and the presented data, the effect of zoledronate on BMD was similar in both osteopenic and osteoporotic women over the years, being statistically significant from the second year for the lumbar spine and from the third year for the femoral neck and total hip. After three years, a dose of 5 mg of zoledronate every 18 months (two doses) in osteopenic women and a dose of 5 mg yearly (three doses) in osteoporotic women increased the BMD similarly; in osteopenic women, a dose of 5 mg of zoledronate every 18 months also increased lumbar spine BMD and total hip BMD after six years (four doses). Evidence comparing zoledronate with alendronate and ibandronate has shown low to very low certainty.

When comparing the present review with others, the findings related to the efficacy were similar to those reported by Sanderson et al., Zhou et al., and He et al.^39,40,41^ The main difference was that the target population included was men, corticosteroid users, and frail women with secondary osteoporosis. Despite these findings, they reported similar results regarding a reduction in fractures and an increase in BMD.

Zoledronate is a well-established option for treating osteoporosis, as recommended in various publications, and the present review highlights its benefits. Although the main evidence for osteopenic women is based on one study, the use of zoledronate (5 mg every 18 months) should be considered in this population.

The AACE recommends alendronate, risedronate, zoledronate, and denosumab as initial therapies for patients at high risk of fracture and teriparatide, abaloparatide, denosumab, romozosumab, or zoledronate for patients at very high risk of fracture and those unable to undergo oral therapy.^4^

The EULAR/EFORT recommends that alendronate and risedronate should be the first-choice agents after fragility fractures in patients older than 50 years and for the prevention of subsequent fractures because of their low cost. It is also recommended that zoledronate or denosumab should be indicated when patients have oral intolerance to bisphosphonates, dementia, malabsorption, and show non-compliance, and anabolic agents are recommended for patients with very severe osteoporosis.^42^

## CONCLUSIONS

Moderate- to high-certainty evidence supports the use of zoledronate (5 mg) annually for three years in postmenopausal women with osteoporosis and 5 mg every 18 months for six years in postmenopausal women with osteopenia to reduce the risk of fractures.

Zoledronate was considered safe and was associated with transient post-dose symptoms. It significantly reduced the P1NP and CTX levels from the sixth month until the third year in osteoporotic women and the sixth year in osteopenic women. In addition, it increased the BMD in all bone segments analyzed after the second dose in osteopenic and osteoporotic women.

## INTRAVENOUS ZOLEDRONATE FOR POSTMENOPAUSAL WOMEN WITH OSTEOPENIA AND OSTEOPOROSIS: A SYSTEMATIC REVIEW AND METANALYSIS

**Peer review information**

The São Paulo Medical Journal thanks Cristiano Augusto de Freitas Zerbini and the other anonymous reviewer for their contribution to the peer-review process of this manuscript.

**Peer review reports**

**Table:**

Reviewer: 1 Dr. Cristiano Augusto de Freitas Zerbini Instituto Sírio-Libanês de Ensino e Pesquisa Núcleo de Reumatologia	
First evaluation	Second evaluation
Recommendation: Accept Comments: Congratulations for this very good article. Additional Questions: Does the manuscript contain new and significant information to justify publication? Yes Does the Abstract (Summary) clearly and accurately describe the content of the article? Yes Is the problem significant and concisely stated? Yes Are the methods described comprehensively? Yes Are the interpretations and conclusions justified by the results? Yes Is adequate reference made to other work in the field? Yes Is the language acceptable? Yes Please rate the priority for publishing this article (1 is the highest priority, 10 is the lowest priority): 2 Length of article is: Adequate Number of tables is: Adequate Number of figures is: Adequate Please state any conflict(s) of interest that you have in relation to the review of this paper (state “none” if this is not applicable): none Rating: Interest: 1. Excellent Quality: 1. Excellent Originality: 2. Good Overall: 1. Excellent	Recommendation: Accept Comments: Very good review. Additional Questions: Does the manuscript contain new and significant information to justify publication? Yes Does the Abstract (Summary) clearly and accurately describe the content of the article? Yes Is the problem significant and concisely stated? Yes Are the methods described comprehensively? Yes Are the interpretations and conclusions justified by the results? Yes Is adequate reference made to other work in the field? Yes Is the language acceptable? Yes Please rate the priority for publishing this article (1 is the highest priority, 10 is the lowest priority): 2 Length of article is: Adequate Number of tables is: Adequate Number of figures is: Adequate Please state any conflict(s) of interest that you have in relation to the review of this paper (state “none” if this is not applicable): None Rating: Interest: 2. Good Quality: 1. Excellent Originality: 2. Good Overall: 2. Good
**Reviewer: 2** **Anonymous** **Did not authorize the publication of the peer review reports**	
**First evaluation**	**Second evaluation**
Recommendation: Major revision	Recommendation: Accept
